# Comparison of the Surface Roughness of CAD/CAM Metal-Free Materials Used for Complete-Arch Implant-Supported Prostheses: An In Vitro Study

**DOI:** 10.3390/biomedicines11113036

**Published:** 2023-11-13

**Authors:** Nataly Mory, Rocío Cascos, Alicia Celemín-Viñuela, Cristina Gómez-Polo, Rubén Agustín-Panadero, Miguel Gómez-Polo

**Affiliations:** 1 Department of Conservative Dentistry and Orofacial Prosthodontics, Faculty of Dentistry, Complutense University of Madrid, 28040 Madrid, Spain; natmory@ucm.es (N.M.); acelemin@ucm.es (A.C.-V.); mgomezpo@ucm.es (M.G.-P.); 2 Department of Nursing and Estomatology, Faculty of Health Sciences, Rey Juan Carlos University, 28922 Madrid, Spain; 3 Department of Prosthetic Dentistry, School of Dentistry, European University of Madrid, 28670 Madrid, Spain; 4 Department of Surgery, Faculty of Medicine, University of Salamanca, 37007 Salamanca, Spain; crisgodent@usal.es; 5 Prosthodontic and Occlusion Unit, Department of Stomatology, Faculty of Medicine and Dentistry, Universitat de València, 46010 Valencia, Spain; ruben.agustin@uv.es

**Keywords:** surface roughness, surface texture, CAD/CAM, high-performance polymers, PEEK, zirconia, thermocycling, complete-arch implant-supported prostheses

## Abstract

The roughness of the intra-oral surfaces significantly influences the initial adhesion and the retention of microorganisms. The aim of this study was to analyze the surface texture of four different CAD-CAM materials (two high-performance polymers and two fifth-generation zirconia) used for complete-arch implant-supported prostheses (CAISPs), and to investigate the effect of artificial aging on their roughness. A total of 40 milled prostheses were divided into 4 groups (*n* = 10) according to their framework material, bio.HPP (B), bio.HPP Plus (BP), zirconia Luxor Z Frame (ZF), and Luxor Z True Nature (ZM). The areal surface roughness “Sa” and the maximum height “Sz” of each specimen was measured on the same site after laboratory fabrication (lab as-received specimen) and after thermocycling (5–55 °C, 10,000 cycles) by using a noncontact optical profilometer. Data were analyzed using SPSS version 28.0.1. One-way ANOVA with multiple comparison tests (*p* = 0.05) and repeated measures ANOVA were used. After thermocycling, all materials maintained “Sa” values at the laboratory as-received specimen level (*p* = 0.24). “Sz” increased only for the zirconia groups (*p* = 0.01). B-BP exhibited results equal/slightly better than ZM-ZF. This study provides more realistic surface texture values of new metal-free materials used in real anatomical CAISPs after the manufacturing and aging processes and establishes a detailed and reproducible measurement workflow.

## 1. Introduction

The surface roughness of dental materials which are in contact with soft tissues plays a crucial role in the accumulation and adhesion of biofilm. Rough surfaces promote increased bacterial adhesion due to their larger surface area and cleaning difficulties [1]. Therefore, materials with low surface roughness are essential to minimize bio-adhesion. Previous articles have reported that the clinical acceptability threshold of roughness for dental materials is 0.2 μm (Ra). Below this level, surface roughness does not significantly impact biofilm formation [2]. The microbiota around dental implants differ from the periodontal microbiota [3]. Consequently, one of the main objectives of surface finishing is to achieve a surface roughness below 0.2 mm by utilizing polishing techniques, devices, and materials [4]. Changes in surface roughness can be caused by a complex and changing humid oral environment, changes in temperature [5], manufacturing process (milling, sintering, sandblasting) [6], or modifications performed with rotatory instruments [7]. Furthermore, surface roughness presents significant implications across various crucial aspects for prosthetic dentistry, including bacterial proliferation [8,9], strength [10], optical properties [11,12], and adhesion [13].

The high demands of aesthetic and biocompatible materials have increased the popularity of metal-free materials in prosthetic dentistry and the main emphasis of developers has been on enhancing their optical and mechanical properties. The gold standard for the manufacturing of complete-arch implant-supported prostheses (CAISPs) involved a metal framework and ceramic veneering. However, in recent years, the evolution of digital technologies and cad/cam materials has led to the development of alternative materials such as high-performance polymers like bio.HPP and new generations of zirconia to improve their aesthetics and mechanical properties [14].

Nowadays, high-performance polymer (HPP) materials have gained significant interest in the field of prosthodontics. This attention is attributed to their advantageous mechanical and biocompatible properties, which enhance their possibility to replace traditional metal frameworks in implant-supported prostheses [15]. BioHPP is a modified PEEK with 20–30% of ceramic fillers, and is a semi-crystalline biopolymer characterized by non-allergenic properties, good biocompatibility, low plaque affinity, excellent stability, and high temperature and chemical resistance, as well as resistance to corrosion [16,17]. It also exhibits high mechanical strength [18], high hardness, low water absorption, and low solubility [5]. Furthermore, PEEK-based materials have a low modulus of elasticity like human bone (4 Gpa) [19], which improves the transmission of chewing pressure, reduces stress, and stimulates the bone modeling around the implants. Additionally, advantages include good polishing properties, good esthetics, low weight, and wear resistance [20]. However, PEEK restorations have a white/dentin opaque color and require veneering with composite resin. Considering the mechanical strength [21] and biocompatibility [22], HPP represent a promising alternative for use as framework material in implant-supported rehabilitations [23].

New generations of zirconia have been introduced to combine the advantages of esthetics and translucency with mechanical properties [24]. Zirconia is a polymorphic material with three crystallographic phases: monoclinic, tetragonal, and cubic [25]. It is present in the monoclinic form at room temperature and when heated its phase transformation occurs to its tetragonal form, and further heating leads to its cubic form. Y-TZP (yttria-stabilized tetragonal zirconia polycrystal) can be of three types based on the yttria content: 3Y-TZP (3 mol%), 4Y-TZP (4 mol%), and 5Y-TZP (5 mol%) [26]. The 3Y-TZP (tetragonal zirconia) is the strongest and 5Y-TZP is the most translucent [27]. Recent developments in Y-TZP materials with a higher yttria content (4–6 mol% yttria) have provided a high translucency and adequate strength. Consequently, clinical indications of monolithic zirconia restorations have expanded to avoid interface fractures in veneer-core structures [28,29]; Luxor Z Frame and Luxor Z True Nature (both 5th generation) represent two new brands of these emerging zirconia materials.

The surface roughness of a material results from marks and grooves left by various factors during fabrication, including tools, abrasive particles, and chemical processes [30]. The areal surface roughness parameter “Sa” calculates average surface roughness and is widely used due to its reliability in minimizing the impact of surface defects. It is a 3D surface parameter analogous to “Ra”, a 2D parameter which measures linear roughness. Additionally, “Sz” represents the maximum height of the selected area and is the sum of the highest peak height and the deepest valley depth. While useful, “Sz” can be influenced by surface flaws like scratches and contamination, as it relies on peak values. Therefore, using both parameters is essential to assess the surface texture of dental prostheses [6]. There is limited information available about the surface roughness of these CAD/CAM materials when comparing laboratory as-received prostheses and their roughness values after artificial aging. Moreover, there is a lack of studies evaluating surface roughness in CAISPs.

While PEEK was previously used as a long-term provisional material in complete-arch implant restorations, the emergence of modified PEEK materials, as well as the introduction of new translucent zirconia, has significantly enhanced their mechanical properties. Consequently, their clinical indications have expanded to include definitive complete rehabilitations. Therefore, it is clinically relevant to evaluate a critical property like surface roughness due to its significant implications across various essential aspects for implant prosthetic dentistry.

The main objective of this in vitro study was to evaluate the surface roughness of four CAD/CAM materials used for complete-arch implant-supported prostheses (CAISPs) after manufacturing laboratory procedures and to investigate the effect of thermocycling on their surface roughness. The following null hypotheses were evaluated: 1. There are no statistically significant differences in surface roughness among all the studied materials after the manufacturing of CAISDP frameworks. 2. No differences are found on the roughness of the aged specimens among the groups. 3. Thermocycling does not affect the surface roughness of the investigated materials.

## 2. Materials and Methods

### 2.1. Sample Size Calculation

A previous pilot study (*n* = 3) was conducted to determine the appropriate sample size. Based on the difference between two independent means (BP and ZF groups) using Sa means and SD values, an effect size of 1.4 was calculated. A sample size of (*n* = 10) specimens per group was calculated using G-power software version 3.1.9.6 (Heinrich-Heine University, Düsseldorf, Germany). A two-tailed analysis with a power of 0.80 and a significance level of α = 0.05 was performed. This sample size was consistent with that used in previous studies [1,4,24,31]. 

### 2.2. Specimen Preparation: Materials, Design, and Laboratory Manufacturing

The high-performance polymers selected for this study were bioHPP dentin shade A2 and bio.HPP Plus White (Bredent GmbH & Co, Senden, Germany). The zirconia materials chosen included fifth-generation zirconia Luxor Z Frame and zirconia Luxor Z True Nature, both in shade A3 (Bredent GmbH & Co, Senden, Germany), which are translucent multilayer zirconia materials developed for the CAD/CAM milling of full-contour restorations. The compositions of the experimental materials are detailed in Table 1.

The Bio.HPP materials present a High Fracture Strength of around 1000–1500 N [18]. Additionally, as specified by the manufacturer, the Luxor Z frame zirconia material exhibits a Fracture Strength of >1050 MPa (previously colored disc) and >1100 MPa (white disc version), while the Luxor Z True Nature features a Flexural Strength of 1100 MPa in the cervical region and 750 MPa in the incisal area.

In the present in vitro study, two computer-aided designs for mandibular complete-arch implant-supported prostheses (CAISPs) were employed: one with a total volume STL, and another with a reduced volume framework STL. These STLs were designed using exocad software version 2.4 (Exocad Plovdiv GmbH, Darmstadt, Germany). A total of 40 specimens (CAISPs) were divided into four groups, with *n* = 10 per group, according to their framework material: group B (bioHPP), group BP (bioHPP Plus), group ZF (Luxor Z-Frame zirconia), and group ZM (Monolithic zirconia, Luxor Z True Nature). 

For the zirconia rehabilitations, the designs were created with approximately 20% enlarged dimensions to compensate for sintering shrinkage. Subsequently, the samples were milled from pre-sintered zirconium oxide discs using computer-aided machining techniques. Following the milling process, the zirconia frameworks were sintered in a furnace Programat P510 (Ivoclar Vivadent, Schaan, Liechtenstein) according to the manufacturer recommendations. The sintering protocol for bridges above 7 units involved heating at 5 °C/min to 900 °C and then at 2 °C/min to 1500 °C, with a 120-min hold time, and then cooling at 3 °C/min to 900 °C and 7 °C/min to 300 °C. The total time was 13–15 h. In group ZF, a veneering ceramic was applied using a layering technique, with low-fusing glass–ceramic IPS e.max^®^ Ceram (Ivoclar Vivadent, Schaan, Liechtenstein) in shade A2. Then, ZF and ZM samples were glazed, and no mechanical polishing was performed. 

**Figure 1:**
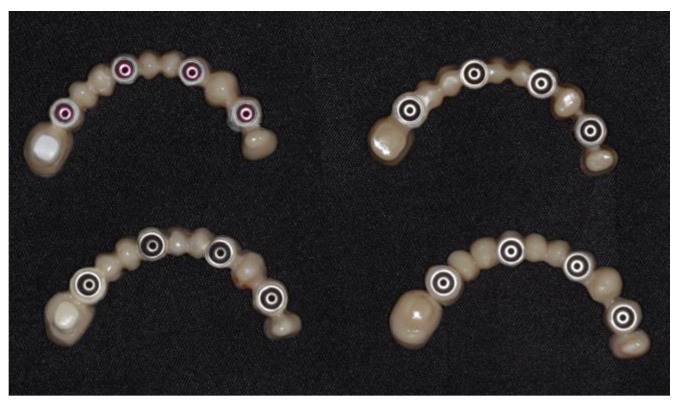
Experimental samples: B (**lower left**); BP (**upper left**); ZF (**lower right**); ZM (**upper right**).

Peek-based rehabilitations were milled from bioHPP and bioHPP Plus Bre.cam disks (98.5 mm diameter and 20 mm thickness) using a dental laboratory milling unit (CORiTEC 350i; imes-icore). Group B and BP specimens were veneered with composite resin crea.lign (Bredent GmbH & Co, Senden, Germany), except in the basal area. Finally, the finishing of specimens was completed with the visio.lign toolkit (Bredent GmbH & Co, Senden, Germany) for bioHPP. All procedures were conducted following the manufacturer’s specifications (Figure 1). The specimens were subject to ultrasonic cleaning in distilled water for 5 min and then dried with compressed air.

### 2.3. Surface Texture Analysis

**Figure 2:**
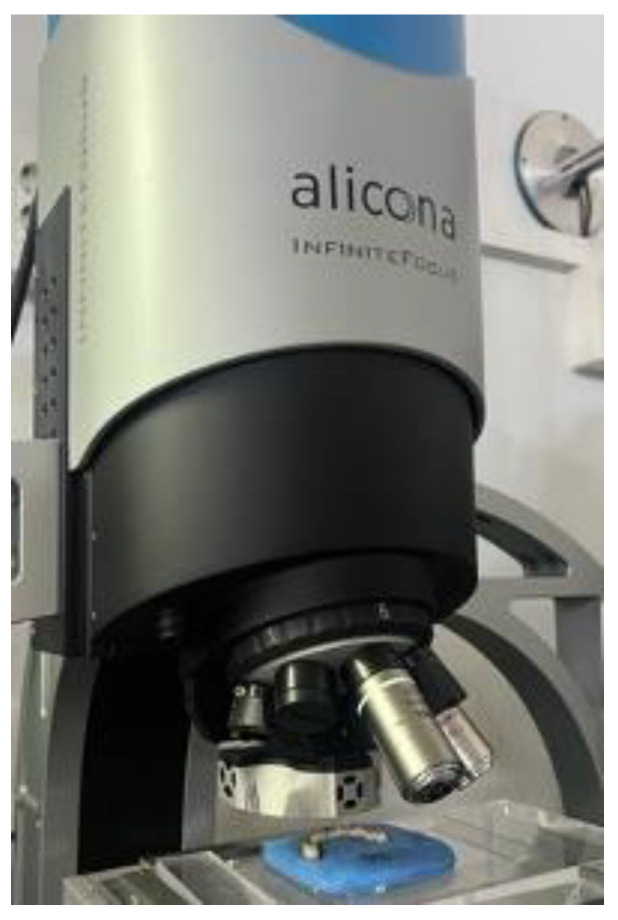
Optical measuring equipment.

The surface texture of each specimen was measured using a non-contact optical profilometer Alicona Infinite Focus XL200 G5 (Alicona Imaging GmbH, Raaba/Graz, Austria) equipped with a measurement software program (MeasureSuite v. 5.3.1; Alicona Imaging GmbH, Raaba/Graz, Austria). For each specimen, an area of 1.62 × 1.62 mm^2^ was analyzed at the center of the basal side of the 4.6 first molar (non-veneered area). This area corresponds to the maximum field of view achievable using the 10× magnification objective. The same point was observed on each specimen with the use of a silicon mold to allow for comparable results (Figure 2). 

**Figure 3:**
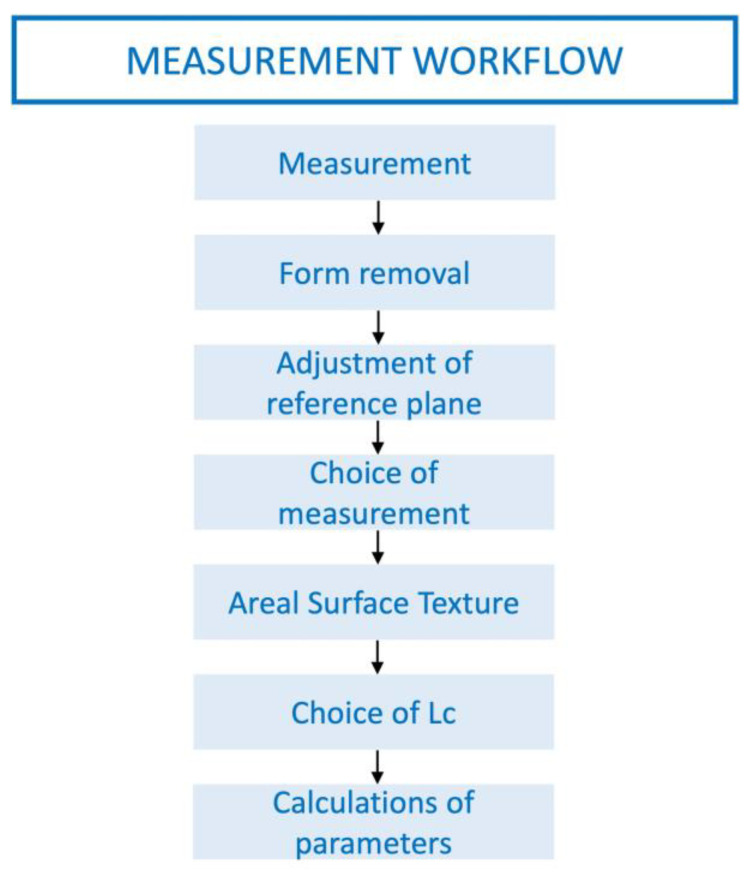
Surface texture measurement workflow.

**Figure 4:**
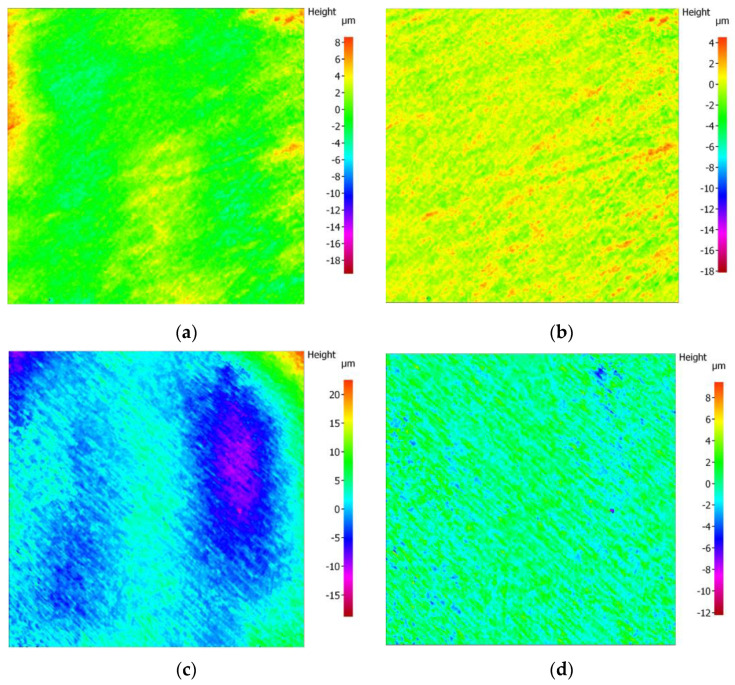
Surface texture measurement: images of the B group (**a**) original dataset; (**b**) filtered roughness dataset; and ZF group (**c**) original dataset; (**d**) filtered roughness dataset at 10× magnification.

A focus variation microscope was chosen as one of the most suitable instruments to perform high-precision three-dimensional surface texture measurements. The surface area was examined in 3D images at 10× magnification. Two tridimensional roughness parameters “Sa” (average areal surface roughness) and “Sz” (maximum height) were evaluated. Specimens were initially measured after laboratory manufacturing “lab as-received specimen”. These measurements were considered as baseline values (Sa, Sz). After the artificial aging procedure, the same parameters were measured for the “aged specimens” (Sa tmcl, Sz tmcl), and values were registered in microns. The measurement workflow applied was conducting following the manufacturer’s instruction for surface texture measurement. This involved the initial form removal, adjustment of the reference plane, application of the roughness filter to separate roughness from waviness, and using a 0.8 mm cutoff, according to ISO 25178 standards [32,33] (Figure 3). A Gaussian filter was used to eliminate tilt from every surface analysis. Color topographic map images represent the height variations on the surface of the samples (Figure 4). Changes in Sa (Sa-Sa tmcl) and Sz (Sz-Sz tmcl) were analyzed to assess the effect of thermocycling on material roughness. 

The accuracy of the profilometer was calibrated for every 10 measurements and all measurements were carried out by a single trained operator. The specimens were codified with an ID number prior to the roughness measurements. The operator conducting the measurements and the statistician performing the data analysis did not have information about the material being evaluated, ensuring a double-blind test.

### 2.4. Hydrothermal Aging Process of Specimens

To simulate artificial aging, all the specimens were thermocycled for 10,000 cycles, equivalent to one year of clinical function [1]. Thermocycling was performed using a thermal cycler device (VA55, Euroortodoncia, Madrid, Spain) in distilled water at temperatures of 5 ± 5 °C and 55 ± 5 °C, with an immersion time of 20 s in each bath and a transfer time of 10 s. After the process, the thermocycled (tmcl) specimens were cleaned ultrasonically in distilled water for 5 min and dried.

### 2.5. Microscopic Observation

A microscopic analysis of two randomly selected samples for each group was conducted (basal side of the 4.6) before and after thermocycling using an optical microscope (VE4, Euroortodoncia, Madrid, Spain) at a magnification of 10×. Images of the samples were captured and subsequently subjected to visual inspection.

### 2.6. Statistical Analysis

All statistical analyses were performed using a statistical software program SPSS Statistics version 28.0.1.1 (IBM Corporation, Armonk, NY, USA). Descriptive statistics, including mean, median, and standard deviation, were calculated. The normality of the data was assessed using the Shapiro–Wilk test, and parametric statistics were applied. Data were analyzed using a two-sided one-way ANOVA, followed by the Bonferroni corrected test for multiple comparisons to determine the effect of material types. A repeated measures ANOVA was employed to assess the effect of the aging process on surface roughness. A significance level of “*p* < 0.05” was stablished. 

## 3. Results

### 3.1. Surface Roughness Measurements

The areal surface texture was determined using two commonly used tridimensional roughness parameters: Sa (average areal surface roughness) and Sz (maximum peak-to-valley height). All the samples were measured before and after thermocycling. 

The descriptive statistics, mean surface roughness values, and standard deviations for the “Sa” and “Sz” parameters are displayed in Table 2.

#### 3.1.1. Baseline Data

After the manufacturing laboratory procedure, the Sa highest values were observed in the ZF (0.75 ± 0.15) and BP (0.75 ± 0.09) groups, followed by the ZM and the B group with (0.70 ± 0.09) and (0.66 ± 0.07), respectively. Regarding the Sz parameter, the highest value was also obtained in the ZF group (33.63 ± 23.91), followed by the ZM (18.98 ± 9.29), B (16.13 ± 5.84), and BP (15.08 ± 3.27) groups. 

**Figure 5:**
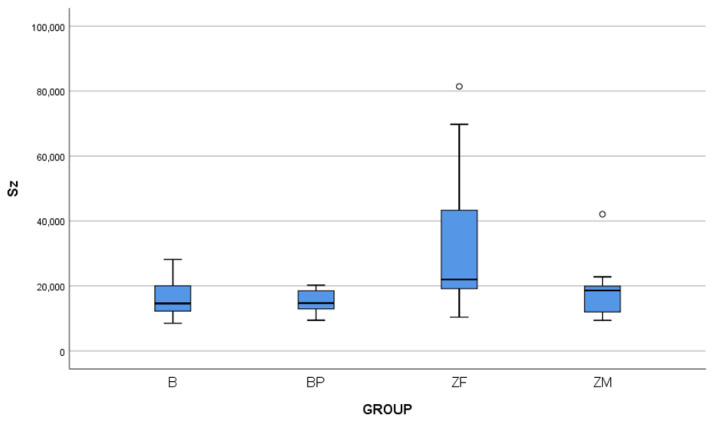
Representative box plot for Sz (μm) baseline data according to total values per group. White dot represents the outlier values.

At the initial Sa values, there were no statistically significant differences among the groups (*p* = 0.21). However, significant differences were found for the Sz parameter (*p* = 0.01). These differences were specific between the ZF group and the B and BP groups, while no significant difference was noted with the ZM group. The results of the Sz measurements are presented in Figure 5.

#### 3.1.2. Data after Aging Process

Thermocycled specimens (5–55 °C, 10,000 cycles) were analyzed. For the Sa tmcl variable, the ZF group had the highest value (0.81 ± 0.14) and the B group (0.65 ± 0.13) the lowest value. However, no significant differences were detected among the four groups (*p* = 0.09). Regarding the Sz tmcl parameter, the highest value was also obtained in the ZF group (56.55 ± 23.92), followed by the ZM (33.85 ± 14.90), B (16.56 ± 11.48), and BP (15.19 ± 4.01) groups. A one-way ANOVA followed by the Bonferroni post-hoc test revealed significant differences between the ZF group and the rest of the groups (B, BP, and ZM) (*p* < 0.05). 

#### 3.1.3. Effect of Thermocycling

**Figure 6:**
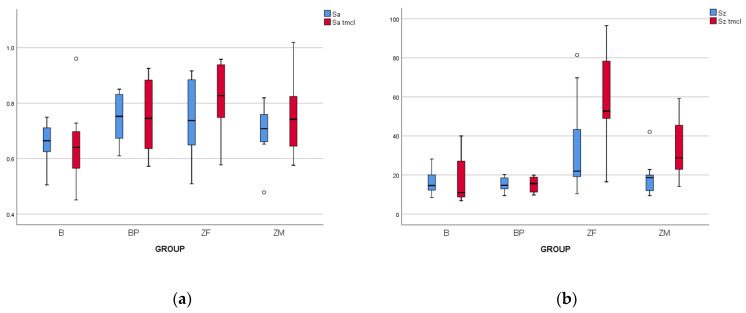
Representative boxplots of the surface parameters (μm) among studied groups. (**a**) Sa (baseline data) and Sa tmcl (after tmcl data); (**b**) Sz (baseline data) and Sz tmcl (after tmcl data). White dot represents the outlier values.

A repeated measures ANOVA test was used to assess the influence of thermocycling on the initial surface roughness. Thermocycling exhibited a minimal effect on the “Sa” parameter in relation to the studied materials. No statistically significant differences were found between Sa and Sa tmcl among the four groups. However, significant differences between Sz and Sz tmcl were observed, exclusively in the zirconia groups (ZF and ZM). Hence, the both Sa and Sz values for HPP groups (B and BP) after aging remained similar to those of the lab as-received specimens. The results of measurements for both 3D roughness parameters, before and after thermocycling, are presented in Figure 6.

### 3.2. Surface Microscopic Analysis

**Figure 7:**
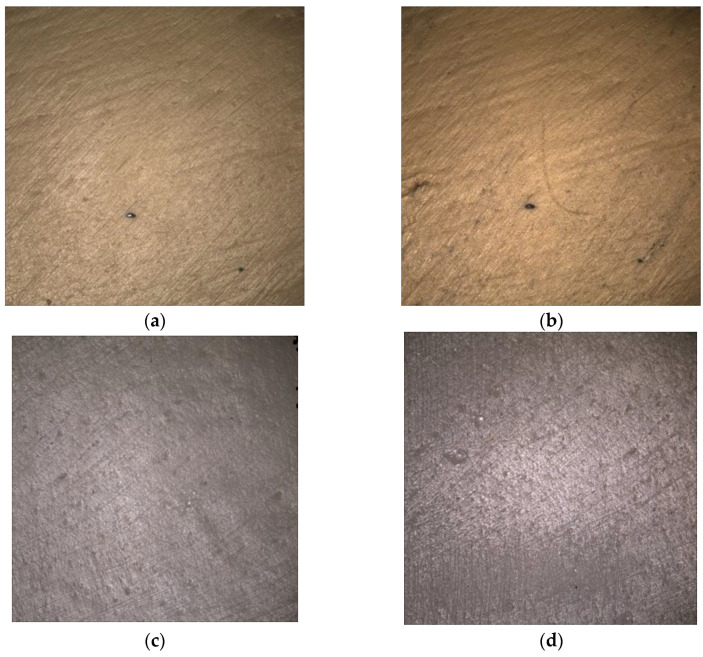
Microscopic images of the B (**a**,**b**) and BP (**c**,**d**) groups at 10× magnification. (**left**) Baseline; (**right**) after tmcl. Small pores and milling lines were observed.

Microscopic assessment at 10× magnification revealed that thermocycling did not induce significant alterations in the modified PEEK-based materials. No cracks were observed, and only small pores and milling lines were detected before and after artificial aging in both the B and BP groups (Figure 7).

**Figure 8:**
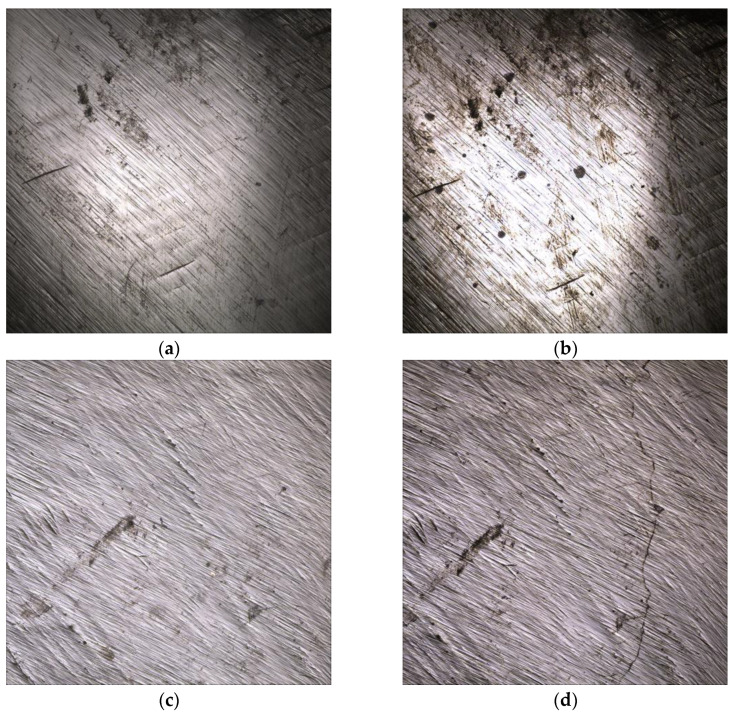
Microscopic images of the ZF (**a**,**b**) and ZM (**c**,**d**) groups at 10× magnification. (**left**) Baseline; (**right**) after tmcl. Scratches and flaws more visible after tmcl.

On the other hand, the zirconia groups exhibited surfaces with minor irregularities, including scratches of the milling bur, pre-existing flaws, and small cracks that became more pronounced after artificial aging (Figure 8).

## 4. Discussion

In this study, we provide novel insights into surface roughness measurement and its significance in prosthetic dentistry. The main objective of this research is to assess the effect of thermocycling on the surface roughness of state-of-the-art materials, including the latest developments in high-performance polymers (bio.HPP Plus) and translucent zirconia materials (Luxor Z Frame and Luxor Z True Nature). Additionally, we aim to introduce a comprehensive method detailing the essential steps for achieving accurate surface roughness measurements within a determined area, with the goal of reducing inaccuracies associated with conventional linear measurements.

Surface roughness is the most important parameter for describing surface texture and plays a crucial role in various clinical aspects. Its assessment is essential due to its direct influence on microbial adhesion and biofilm formation [1]. This study aimed to compare the surface roughness of framework materials for CAISPs. Previous studies have reported similar results for average roughness in bioHPP [4] and 5Y-ZP zirconia specimens [24]. However, this current research explored differences in specific 3D parameters, “Sa” (average roughness) and “Sz” (highest peak to valley) to achieve a better understanding of surface texture.

Initially, our results indicated that there were no significant differences in the baseline data among the groups for the Sa parameter, suggesting that the materials exhibited similar surface properties. However, we found statistically significant differences for the Sz parameter between the ZF group and the B-BP groups, but not with the ZM group. As a result, the first null hypothesis was partially rejected. These statistical differences between the ZF and B-BP groups can be attributed to the different composition of polymers and ceramics [9].

Moreover, manufacturing procedures can influence surface roughness. Notably, only the ZF group showed significant differences in Sz value, which can be partly attributed to an additional firing process for ceramic veneering during the fabrication of dental prostheses [7], while the ZM group lacks such a coating. It is also possibly due to increased milling complexity and greater flexural strength and hardness compared to the ZM group [34]. Standard deviations (SD) in Sz are notably higher, displaying considerable variability, as is commonly observed in studies evaluating Sz [35]. The zirconia groups showed higher SD compared to the PEEK groups, with the ZF group registering the highest value.

Additionally, the microscopic analysis revealed that ZF exhibited different surface characteristics compared to B and BP, showing more surface defects. These defects may be a result of larger grains within the material, which are removed during the milling or adjustment process [36]. 

In the aged specimens, Sa tmcl values showed no differences, but significant differences in Sz tmcl were observed for the ZF group in comparison to the other groups. This could be because the ZF group had a composition that differs from the B-BP group, as well as the ZM group. ZF has a higher ZrO_2_ content and lower yttria content compared to ZM. These slight variations may make it more susceptible to a phenomenon known as low-temperature degradation (LTD), a chemical property of zirconia that results in surface degradation, microcracking, and reduced strength in the presence of humidity [37]. 

Considering the effect of thermocycling (TMCL), the results of Sa confirm that it does not increase average roughness for PEEK-based and zirconia materials. In contrast, both zirconia groups (ZF-ZM) showed intra-group differences between initial and post-TMCL values. This can be explained by the zirconia’s susceptibility to LTD, which involves a slow transformation from the tetragonal (t) to monoclinic (m) phase, induced by thermal or mechanical stress, and exacerbated in the presence of water, causing a surface deterioration [38]. The presence of Al_2_O_3_ stabilizes the tetragonal phase and improves hydrothermal ageing resistance; therefore, when the alumina content decreases (≤0.05 wt.%), zirconia is more translucent but more susceptible to LTD [39]. Conversely, a higher yttria content reduces LTD by producing a less monoclinic phase and providing greater structural stability. Consequently, translucent zirconia (5Y-ZP) exhibits lower susceptibility to LTD than tetragonal zirconia (3Y-TZP); however, 3Y-TZP still has an adequate durability in oral conditions [40,41]. Theoretically, the aging process should lead to an increase in surface roughness. However, when comparing the initial and final roughness of the bioHPP samples, it became evident that the B-BP groups did not show significant changes in the Sa and Sz parameters, and their values for Sa-Sa tmcl and Sz-Sz tmcl remained unaltered. Notably, the aging process in the zirconia groups ZF-ZM led to significant differences in the Sz-Sz tmcl parameters, with both groups also displaying the highest initial Sz values. These variations in Sz are indicative of the presence of pores, cracks, and flaws, making it a valuable parameter for assessing surface irregularities and specific defects.

In recent years, numerous studies have evaluated surface roughness in PEEK and zirconia materials [2,4,5,7,8,9,24]. However, only one has been conducted on samples with real anatomies (monolithic zirconia 3-unit bridges) [11]. These studies reveal two distinct trends: some report more favorable results for zirconia materials [22,42], while another opposing trend observes lower roughness in PEEK materials [8]. In thermocycling-induced artificial aging, most studies do not report significant differences in average roughness values (Sa, Ra) before and after TMCL for the two main material types assessed in this study: HPP [1] and translucent zirconia [26]. 

In most literature reports [4,7,22,24,31], surface texture is typically assessed using simple profile surface measures like Ra or Rz, while areal parameters have proven to be more informative [6]. However, despite using the same parameter, results vary widely, possibly due to the use of different measurement equipment. Some studies use contact profilometers, which provide R values (a line), while others use non-contact optical systems (interferometric, focus variation, or confocal microscopes, AFM), which offer S values (an area). Non-contact optical systems can also calculate bidimensional parameters but may not fully represent the surface. The methodological differences and the wide range of parameter values are mainly attributed to the measurement methods and procedures selected. The absence of standardized measurement methods and failure to specify key factors, such as the length of the profile path for Ra or the cutoff filter, further complicate their reproducibility. In our research, measurements were conducted at a 10× magnification using a Gaussian filter and a cutoff length of 0.8 mm, following ISO 25178 standards. While numerous methods are available for measuring surface roughness, there is a lack of standardized procedures for conducting comparisons across different studies. ISO 4287:1999 [43] measures linear roughness “Ra”, which is limited to 2D measurement, while the more recent ISO 25178-2:2012 includes updated terms, definitions, and parameters for 3D evaluation [33]. “Sa”, a 3D parameter, offers more comprehensive data by measuring entire areas, rather than just linear profiles. Sa values are also influenced by magnification, with higher magnification yielding lower roughness values [30]. Although the values obtained for Sa exceeded the clinically acceptable limit of Ra = 0.2 μm [2], we could not directly correlate 3D–2D parameters, as they are not equivalent.

In the realm of laboratory processing, ceramics have been categorized as difficult-to-machine materials due to their high hardness and brittleness. After milling, tiny cracks may develop on the final surface [6]. Also, 5Y-ZP did not undergo transformation toughening and as a result may not be as tolerant to the surface damage introduced during the fabrication (milling, sintering), adjustment, and airborne particle abrasion of a zirconia restoration [34]. Sintering conditions have a strong impact on stability and mechanical properties, and affects surface texture. The type of sintering (traditional or fast) affects the properties. Novel speed sintering protocols have been developed to meet the demand for time and cost-effective CAD/CAM restorations [44]. Wertz et al. reported that the milling process may increase the monoclinic phase after machining, but it disappears after sintering. Moreover, glazing and sandblasting processes have minimal influence on the crystallographic structure phases [45]. However, an increase in roughness (Sa, Sz) was observed after sandblasting [46]. Kim et al. reported that translucent zirconia requires 50 µm alumina sandblasting to prevent surface damage [47]. In some cases, differences in surface roughness may not be detected using only average roughness parameters, Sa, or Ra. When this occurs, it is essential to focus on other parameters such as Sz, which allows us to assess the unevenness. In a previous study conducted by Fernández et al., the Sz parameter was evaluated in milled Cr-Co frameworks, yielding a value of 29 µm, which is comparable to the initial Sz value observed in our study for the ZF group (33 µm) [35].

This study has the typical limitations of in vitro studies. Mechanical and biological factors, such as occlusion and the oral environment, were not replicated, which limits the direct extrapolation of the data to the clinical situation. Furthermore, bacterial adhesion was not evaluated, which could have provided additional insights regarding the relationship between surface roughness and bacterial adhesion. Additionally, comparing different roughness measurement equipment was not feasible due to a lack of reported details in previous studies, highlighting the need to consider these details in future investigations.

While, traditionally, metal–ceramic prostheses have been the primary choice for CAISPs, demonstrating high clinical performance and long-term survival rates, it is important to consider the potential impact of alternative materials like zirconia and HPP, which offer biocompatibility and optimal mechanical properties. Moreover, HPP has the ability to absorb occlusal forces [48], making it interesting to evaluate its damping effects on masticatory function in order to prevent the overloading of implants and temporomandibular disorders. Ultimately, the extensive literature on surface roughness has revealed correlations with other parameters such as flexural strength [7,10], antagonist wear [49], translucency [50], etc., which could be explored in future research. 

Further comparative clinical studies are essential to validate the utilization of these new materials and determine their performance and applications in clinical practice.

## 5. Conclusions

Considering the limitations inherent to the present in vitro study, the following conclusions were drawn:

The thermocycling process did not show a statistically significant impact on the average areal surface roughness (Sa) of the CAD/CAM studied materials, including high-performance polymers and fifth-generation zirconia. Statistically significant differences were only found for the maximum peak–valley heigh (Sz) in the zirconia groups.

Furthermore, it is noteworthy that the surface roughness observed in BioHPP (modified PEEK) groups closely resembled that of zirconia.

Significantly, microscopic observations did not reveal the presence of microcracks in the surface texture of the B and BP groups. 

## Figures and Tables

**Table 1:** Summary of experimental materials, abbreviations groups, compositions, and brand names.

Framework Material	Groups	Chemical Composition	Brand Name
Polyether ether ketone	B	Polyether ether ketone with 20 wt.% inorganic fillers *	Bio.hpp
Polyether ether ketone	BP	Polyether ether ketone with 25 wt.% inorganic fillers *	Bio.hpp Plus
Zirconia frame	ZF	94–95% ZrO_2_, 4.5–5.5% Y_2_O_3_, <0.5% Al_2_O_3_, <0.5% other oxide	Luxor Z Frame
Zirconia monolithic	ZM	90–95% ZrO_2_, 4–10% Y_2_O_3_, ≤0.5% Al_2_O_3_, <0.5% other oxide	Luxor Z True Nature

* TiO_2_ and inorganic pigment.

**Table 2:** Mean surface roughness values ± SD (μm) for “Sa” (on the left) and “Sz” (on the right), and after manufacturing (baseline) and after thermocycling (tmcl).

Experimental Group	Sa		Sa	
	Baseline	tmcl	Baseline	tmcl
B	0.66 ± 0.07 ^aA^	0.65 ± 0.13 ^aA^	16.13 ± 5.84 ^aA^	16.56 ± 11.48 ^aA^
BP	0.75 ± 0.09 ^aA^	0.75 ± 0.14 ^aA^	15.08 ± 3.27 ^aA^	15.19 ± 4.01 ^aA^
ZF	0.75 ± 0.15 ^aA^	0.81 ± 0.14 ^aA^	33.63 ± 23.91 ^bA^	56.55 ± 23.92 ^bB^
ZM	0.70 ± 0.09 ^aA^	0.75 ± 0.14 ^aA^	18.98 ± 9.29 ^abA^	33.85 ± 14.90 ^aB^

The same superscript lowercase letters in the same column and uppercase letters in the same row indicate no significant differences (*p* < 0.05). See Table 1 for group abbreviations.

## Data Availability

The data presented in this study are available on request from the corresponding author.
